# The Chronic Protective Effects of Limb Remote Preconditioning and the Underlying Mechanisms Involved in Inflammatory Factors in Rat Stroke

**DOI:** 10.1371/journal.pone.0030892

**Published:** 2012-02-08

**Authors:** Dingtai Wei, Chuancheng Ren, Xiaoyuan Chen, Heng Zhao

**Affiliations:** 1 Department of Neurosurgery, Stanford University, Stanford, California, United States of America; 2 Stroke Center, Stanford University, Stanford, California, United States of America; 3 Department of Radiology, Tianjin Medical University General Hospital, Tianjin, China; 4 Department of Radiology, Fujian Medical University Ningde Hospital, Fujian, China; 5 Shanghai No.5 Hospital, Fudan University, Shanghai, China; 6 Laboratory of Molecular Imaging and Nanomedicine (LOMIN), National Institute of Biomedical Imaging and Bioengineering (NIBIB), National Institutes of Health (NIH), Bethesda, Maryland, United States of America; University of Cambridge, United Kingdom of America

## Abstract

We recently demonstrated that limb remote preconditioning (LRP) protects against focal ischemia measured 2 days post-stroke. Here, we studied whether LRP provides long-term protection and improves neurological function. We also investigated whether LRP transmits its protective signaling via the afferent nerve pathways from the preconditioned limb to the ischemic brain and whether inflammatory factors are involved in LRP, including the novel galectin-9/Tim-3 inflammatory cell signaling pathway, which induces cell death in lymphocytes. LRP in the left hind femoral artery was performed immediately before stroke. LRP reduced brain injury size both at 2 days and 60 days post-stroke and improved behavioral outcomes for up to 2 months. The sensory nerve inhibitors capsaicin and hexamethonium, a ganglion blocker, abolished the protective effects of LRP. In addition, LRP inhibited edema formation and blood-brain barrier (BBB) permeability measured 2 days post-stroke. Western blot and immunostaining analysis showed that LRP inhibited protein expression of both galectin-9 and T-cell immunoglobulin domain and mucin domain 3 (Tim-3), which were increased after stroke. In addition, LRP decreased iNOS and nitrotyrosine protein expression after stroke. In conclusion, LRP executes long-term protective effects against stroke and may block brain injury by inhibiting activities of the galectin-9/Tim-3 pathway, iNOS, and nitrotyrosine.

## Introduction

Remote preconditioning refers to a brief or repeated brief ischemia performed in a distal organ to protect against a prolonged ischemia in another vital organ. Limb remote preconditioning (LRP), which is performed in hind limbs, is one of the most frequent remote preconditioning methods used to protect against heart and brain ischemia. LRP was reported to reduce hippocampal neuronal injury in global ischemia in rats [1], and this has been confirmed by several independent research groups using cardiac arrest or global ischemia models [2]–[4]. In addition, we recently showed that LRP performed immediately, 12 hours and 2 days before stroke reduced infarct size measured 2 days post focal cerebral ischemia [5]. In terms of clinical applicability for stroke treatment, remote preconditioning may have advantages over conventional ischemic preconditioning because instead of the higher risk treatment directly to the brain, remote preconditioning is performed in a non-vital organ [6]–[8]. In fact, remote preconditioning has moved into clinical trials in carotid endarterectomy [9], where preconditioning with 10 minutes of lower limb ischemia-reperfusion tended to improve neurological deficits as measured by saccadic latency, although no significant difference was reached. To facilitate the translation of remote preconditioning to stroke patients, more studies are needed to understand its underlying protective mechanisms.

Previously we reported on the short-term protection of LRP on infarct size [5]. Here we further address whether LRP has long-term protective effects. Because some neuroprotectants only offer transient protection against brain injury [10], [11], we measured the size of brain injury and performed behavioral testing for up to 2 months. Edema formation and blood brain-barrier (BBB) permeability are critical to brain injury; therefore, we also measured the effects of LRP on these factors. In addition, we studied how limb ischemia transfers protective signaling from the limb to the brain. Since the afferent nerve pathways have been shown to contribute to remote preconditioning in myocardial ischemia [12]–[14], we tested whether these pathways are also involved in the protective effects of LRP in stroke.

Many cell signaling pathways are involved in neuronal death induced by stroke. Nevertheless, limb ischemia may first affect blood circulation and the function of various blood cells, including T cells, macrophages and neutrophiles, which are closely associated with brain inflammation [15]–[17]. Thus, we assumed that LRP may inhibit inflammation in the brain, and chose to investigate whether LRP blocks several molecules critical to the generation of inflammation and free radicals, including galectin-9 and T-cell immunoglobulin domain and mucin domain 3 (Tim-3), inducible nitric oxide synthase (iNOS), and nitrotyrosine.

Tim-3 regulates the inflammatory response [18]. When Tim-3 is expressed on CD4^+^T-helper 1 (T_H_1) cells, it is triggered and activated by its ligand galectin-9, which causes calcium influx and cell aggregation that induces T_H_1 cell death. Therefore, it inhibits the inflammatory response by eliminating T_H_1 cells [19]. In addition, Tim-3 is also expressed on nerve cells in the brain [20], but its role in the brain is not known.

This study used one of our previously established LRP model in rats [5] where LRP was performed immediately before stroke onset. We used a battery of behavioral tests to examine neurological deficiency for up to 2 months and used Western blot and immunostaining to investigate protein levels of the inflammatory and oxidative factors mentioned above.

## Results

### LRP offered long-term protection and attenuated neurological deficiency

**Figure 1:**
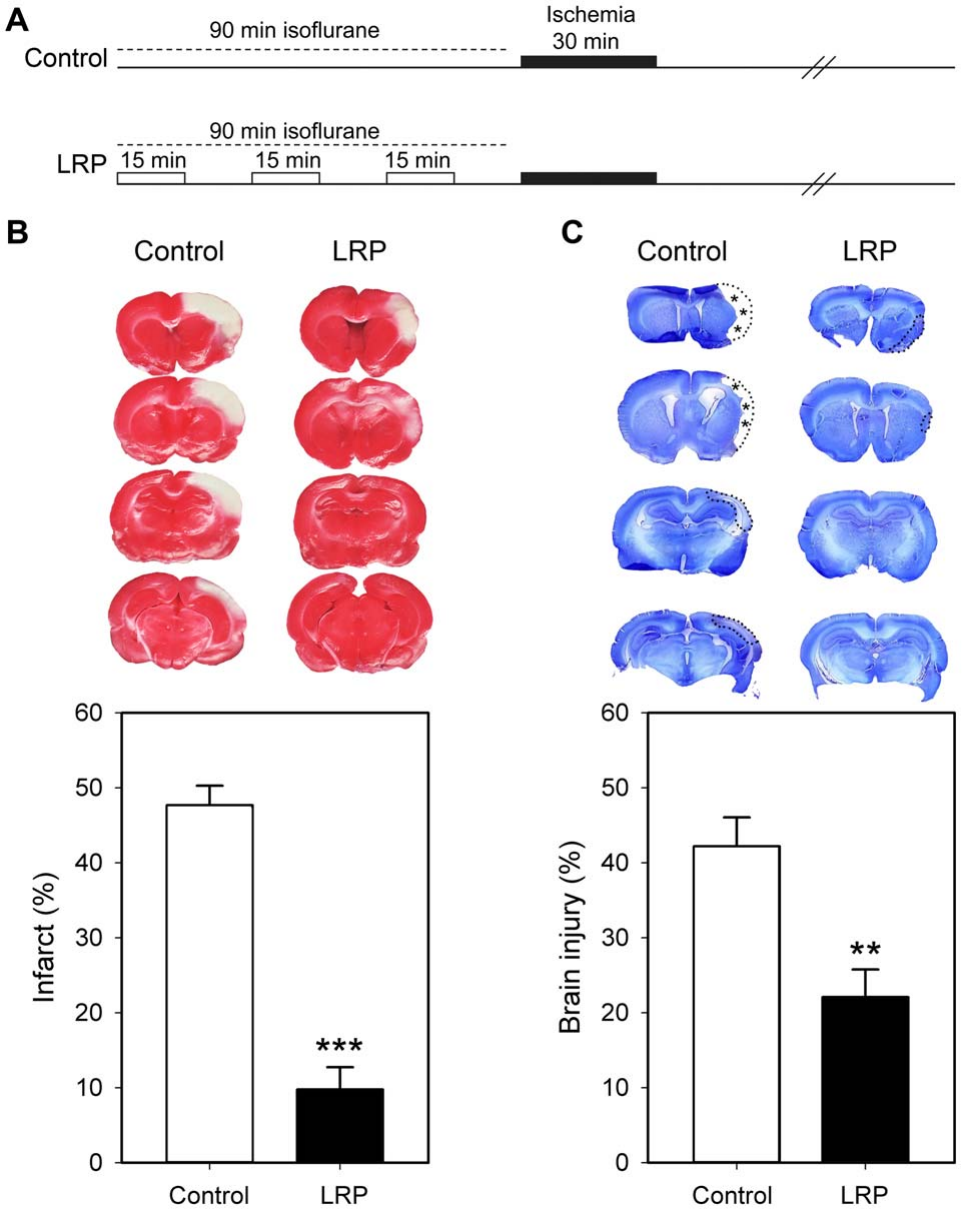
LRP reduced brain injury after focal ischemia. **A.** Diagram of the LRP protocol. In the LRP group, 3 cycles of 15 min occlusion/reperfusion of the left femoral artery was induced before stroke onset. In the control group, 90 min of isoflurane was applied before ischemia, as a vehicle control for LRP. **B.** Top: Representative brain sections of TTC staining from rats receiving focal ischemia with and without LRP. Bottom: Bar graph showing the quantitation of infarct sizes in the ischemic cortex measured at each level and normalized to the non-ischemic contralateral cortex, and expressed as percentage. **C.** Top: Representative staining of cresyl/violet from rat brains 60 d post-stroke. The lost and damaged tissues are traced with dashed lines. Bottom: Bar graph showing the average value from 4 levels of brain sections. Control, control ischemia. LRP, limb remote preconditioning. N = 6–7/group. **, ***, *P*<0.01, 0.001, respectively, vs. control.

**Figure 2:**
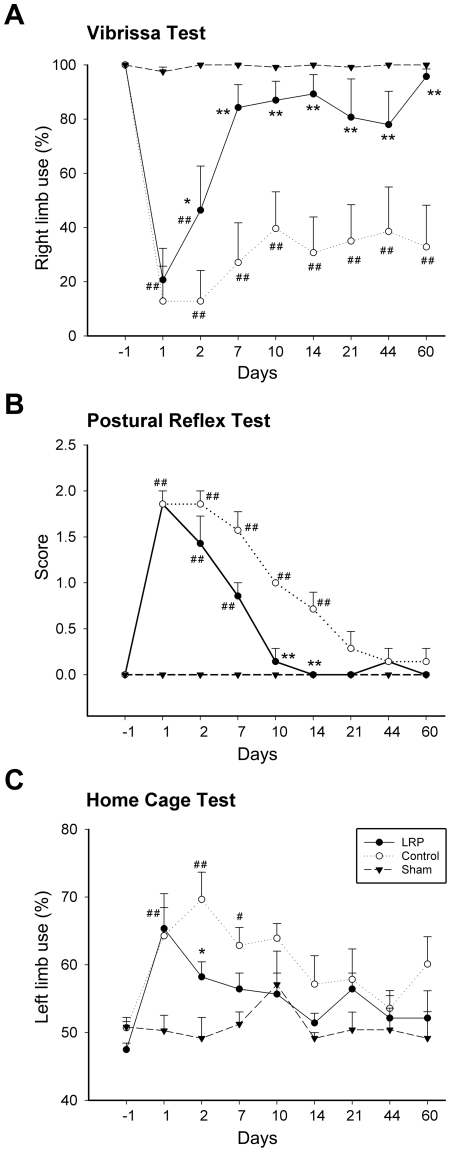
LRP attenuated behavioral deficits for up to 2 months post-ischemia. Three standard tests were performed. **A.** Vibrissae-elicited forelimb placement test. All sham rats showed normal forelimb placing. Control ischemic rats exhibited unsuccessful placing of the contralateral forelimb (right) after stroke. The reflex was tested 10 times on each side per trial, and 2 trials occurred per test session. The percentage of vibrissa stimulations in which a paw placement occurred was calculated. LRP attenuated the overall deficit from 2 to 60 d after stroke. **B.** Postural reflex test. Scores were increased in control rats at 1, 2, 7, 10, and 21 d after stroke; LRP reduced scores at 10 and 14 d after stroke compared with control stroke. **C.** Home cage forelimb use test. The number of times the animal used its forelimbs to brace itself against the wall of the cage was counted, with separate counting for the ipsilateral, contralateral, or both forelimbs until 20 contacts were reached. The percentage of times out of 20 that the ipsilateral forelimb (left) was used was computed. The ratio of left-limb-use was increased at 1, 2 and 7 d compared to control ischemia; LRP blocked this increase at 2 d. *, ** vs. control ischemia, and #, ##, vs. sham, *P*<0.05, 0.01 at the corresponding time points, respectively. N = 6–8/group.

LRP performed immediately before ischemia onset reduced infarct size measured 2 days post-stroke (Fig. 1B) as we previously reported [5]. In this study, we further detected long-term protective effects of LRP in both brain injury and behavioral tests. Histological results demonstrated that LRP reduced ischemic injury size by 40% at 2 months after stroke (Fig. 1C). Three standard behavioral tests were performed to evaluate the protective effect of LRP on neurological function (Fig. 2). In the vibrissae test, placing of the forelimb contralateral to the injury was disrupted from 1 to 60 days after stroke in control ischemic rats; LRP did not prevent such disruption at 1day but attenuated it thereafter until day 60 (Fig. 2A). Scores for the postural reflex test were increased at 1 and 2 days, remained high until 14 days, then gradually decreased to normal levels in control rats; the scores were attenuated by LRP from 2 to 14 days (Fig. 2B). Lastly, in the home cage test, control ischemic rats showed a bias in favor of the ipsilateral forelimb from 1 to 7 days after stroke; LRP attenuated this bias at 2 and 7 days (Fig. 2C).

### LRP reduced BBB leakage and brain edema

**Figure 3:**
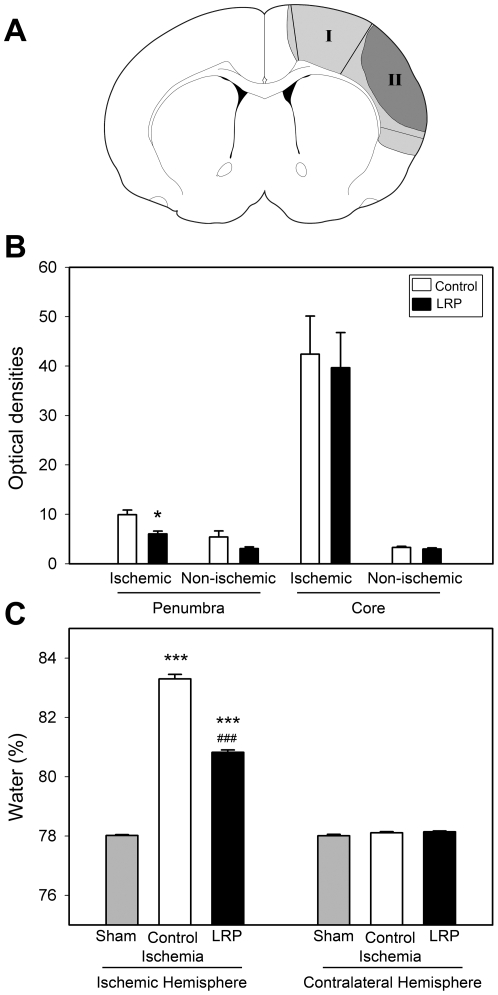
LRP attenuated edema induced by stroke. **A**. Diagram of the dissected regions of the ischemic penumbra and core for BBB leakage measurement. The penumbra (I) is defined as the ischemic region spared by LRP, while the core (II) is the ischemic part that developed into the infarction. The same regions were also dissected for Western blotting. **B**. LRP inhibited BBB leakage. Evans blue was injected 2 h before the rat was euthanized. The ischemic penumbra and core, as well as the corresponding non-ischemic hemisphere were dissected for Evans blue detection. LRP reduced BBB leakage at 48 h after stroke in the penumbra but not in the core (n = 6/group). * vs. control ischemia, *P*<0.05. **C.** LRP mitigated edema after stroke. The ischemic and non-ischemic hemispheres from each rat brain were separated, weighed for wet weight, baked at 90±2°C for 1 wk, and weighed again for dry weight. Water contained in the brain tissues was calculated and is presented in the bar graph (n = 6–7/group). *** vs. contralateral hemisphere, *P*<0.001; ### vs. sham and control ischemia, *P*<0.001.

BBB permeability at 2 days post-stroke was measured by Evans blue. The level of Evans blue was robustly increased at 48 hours after stroke in the ischemic penumbra (Fig. 3A), but much higher levels were detected in the core than in the penumbra. LRP blocked BBB leakage in the penumbra at 48 hours but had no effect in the core (Fig. 3A).

Brain edema was also detected 2 days after stroke by the wet-dry method. More water content was detected in rats receiving stroke with and without LRP than in sham rats without ischemia. However, LRP significantly reduced water content compared to control ischemic rats (Fig. 3B), suggesting that LRP attenuated stroke-induced edema.

### Blocking the nerve pathways inhibited the protective effect of LRP

**Figure 4:**
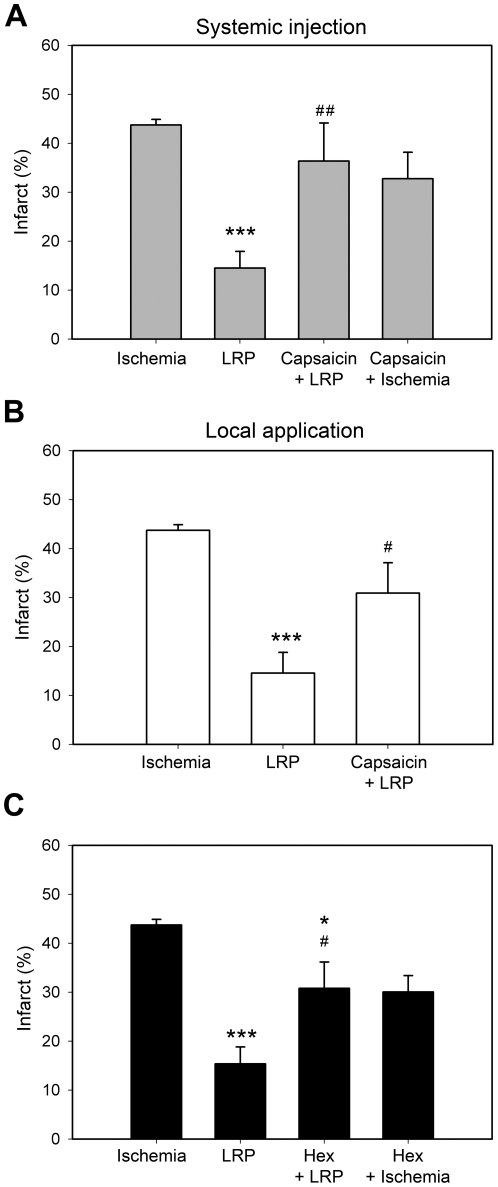
Blocking nerve pathways enlarged infarct size in rats receiving LRP. A. Systemic injection of capsaicin enlarged infarction in rats receiving LRP. Capsaicin was subcutaneously injected into rats for 4 consecutive days. LRP and focal ischemia were conducted 2 wks after capsaicin injection, and infarct sizes were measured 2 d after stroke. The bar graphs represent the mean values of infarct size in 4 groups: 1) ischemia, control ischemia; 2) LRP, animals receiving LRP plus ischemia; 3) capsaicin+LRP, animals receiving capsaicin injection, LRP and ischemia; 4) capsaicin+ischemia, animals receiving capsaicin and control ischemia. **B.** Local application of capsaicin onto the thigh nerve in the hind limb abolished the protective effects of LRP. The nerve was soaked with a capsaicin solution for 30 min. Four days later LRP and focal ischemia were conducted. The bar graphs show average infarct sizes. **C**. The ganglion blocker hexamethonium blocked the protective effects of LRP. Hexamethonium was intravenously injected into rats 30 min before LRP induction. Infarct sizes were measured at 2 days after stroke. The bar graphs show the average values of infarct size of 4 groups. 1) Ischemia, control ischemia without LRP; 2) LRP, animals receiving ischemia and LRP; 3) Hex+LRP, animals receiving hexamethonium, LRP and ischemia; 4) Hex+ischemia, animals receiving hexamethonium and ischemia without LRP. N = 7/group. *, *** vs. ischemia, *P*<0.05, 0.001, respectively. #, ##, vs. LRP.

We examined whether afferent nerve pathways transfer the protective signaling from the preconditioned limb to the brain. First, both systemic injection and local application of capsaicin to the femoral nerve increased the infarct size in rats receiving LRP (Fig. 4A), but systemic injection of capsaicin did not increase infarct size in rats receiving control ischemia. These results suggest that sensitive nerve activity contributes to the protective effects of LRP but had no effect on ischemia alone. Second, we injected the ganglion blocker, hexamethonium, into rats. Again, hexamethonium injection did not enlarge infarct size in rats receiving control ischemia, but it increased infarct size in rats receiving stroke with LRP (Fig. 4B), suggesting that hexamethonium abolished the protective effects of LRP.

### LRP blocked upregulation of galectin-9/Tim-3 expression induced by stroke

**Figure 5:**
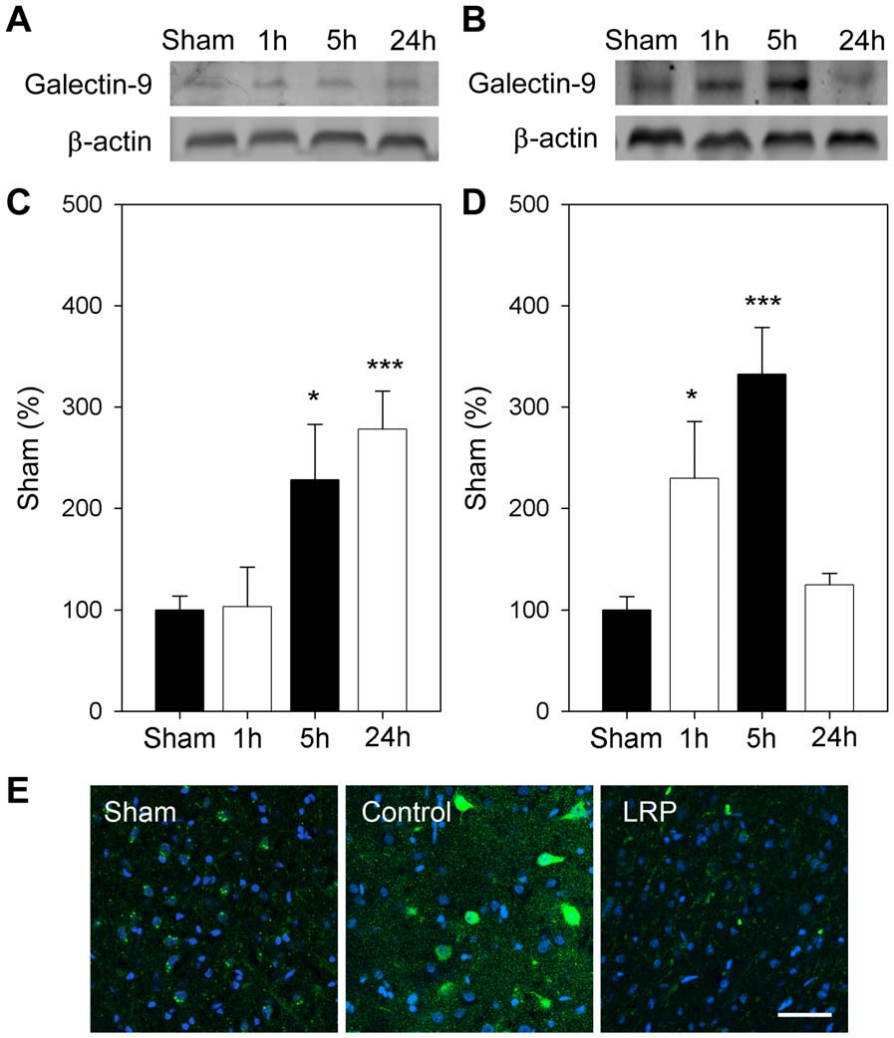
LRP inhibited galectin-9 expression 24 h after stroke. **A and B.** Representative protein bands of galectin-9 from Western blots for control ischemia and LRP with ischemia, respectively. **C and D**. The bar graphs show protein band quantitation results corresponding to A and B, respectively. Brain tissue from ischemic penumbras were dissected for Western blotting, as indicated in Fig. 3A. *, vs. sham, *P*<0.05, ***, vs. sham, *P*<0.001. n = 6/group. **E**. Results from confocal microscopy indicate that galectin-9 was increased in the ischemic penumbra 24 h after stroke, and such expression was inhibited by LRP. Scale bar, 50 µM.

**Figure 6:**
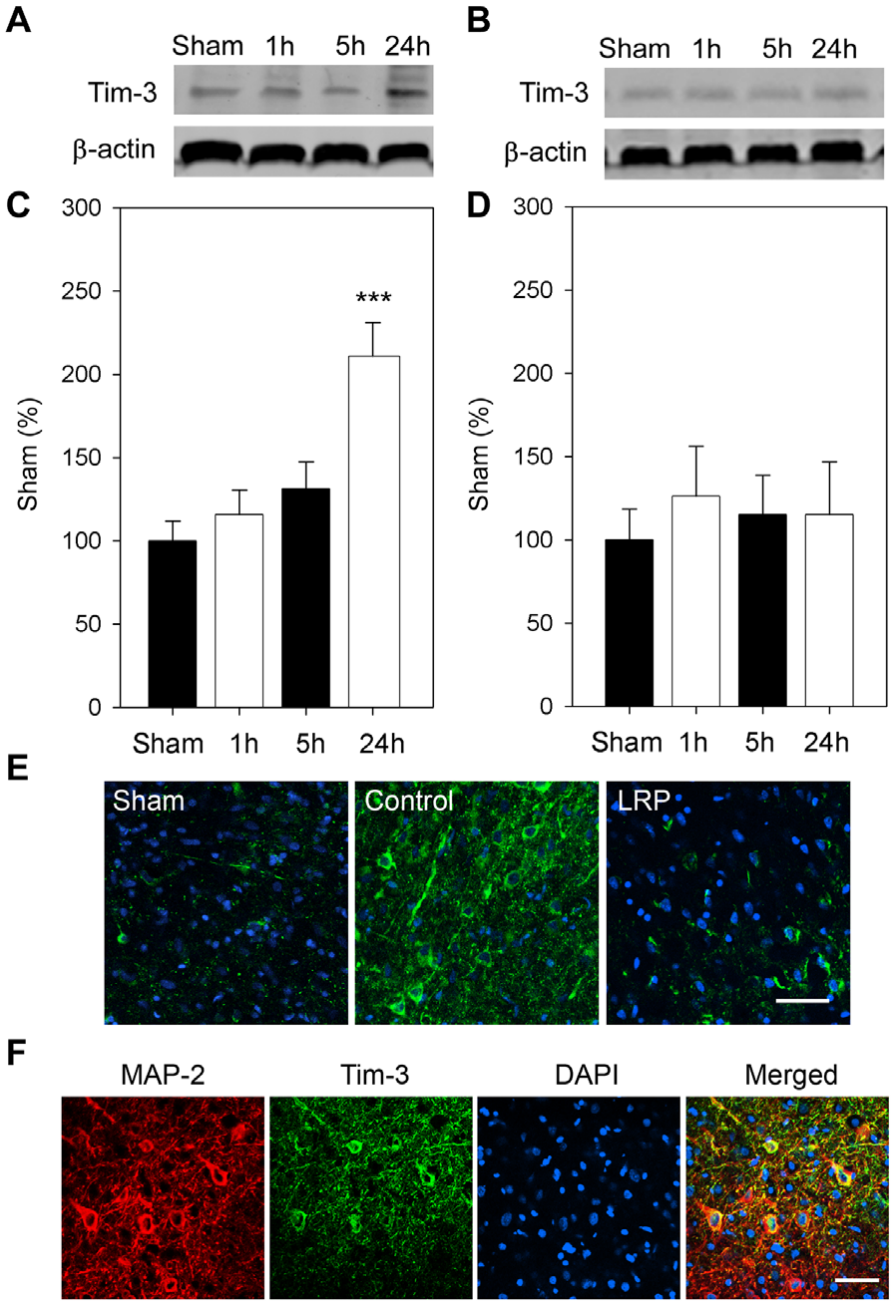
Tim-3 expression was increased after stroke and inhibited by LRP. **A and B.** Western blots showing representative protein bands of Tim-3 and β-actin in the ischemic penumbra for rats receiving control ischemia alone and ischemia plus LRP, respectively. **C.** Bar graphs indicating that Tim-3 was slightly increased as early as 1 h and peaked at 24 h after stroke. **D.** This was inhibited by LRP. ***, vs. sham, *P*<0.001. n = 6/group. **E**. The results were further confirmed using immunofluorescent confocal microscopy in control ischemia and LRP 24 h after stroke. An ischemic brain was collected from a surviving rat 24 h after stroke, fixed for 24 h with 4% PFA, stained, and examined with confocal microscopy. **F.** Double staining of MAP-2 and Tim-3 suggests that Tim-3 was expressed in neurons. Scale bar, 50 µM.

We then investigated whether the novel galectin-9/Tim-3 cell signaling pathway, which causes T_H_1 cell death in the immune system, is involved in neuronal death after stroke. Results by Western blot suggest that the expression of galectin-9 was increased as early as 5 hours after stroke, and lasted at least to 24 hours (Fig. 5). LRP unexpectedly increased the expression of galectin-9 at 1 hour but blocked it at 24 hours. The expression of galectin-9 in control ischemia and the effect of LRP were confirmed by immunofluorescent staining (Fig. 5). Tim-3 expression, which is downstream of galectin-9, was increased only at 24 hours after stroke, and this increase was inhibited by LRP (Fig. 6).

### LRP blocked protein expression of iNOS and nitrotyrosine

**Figure 7:**
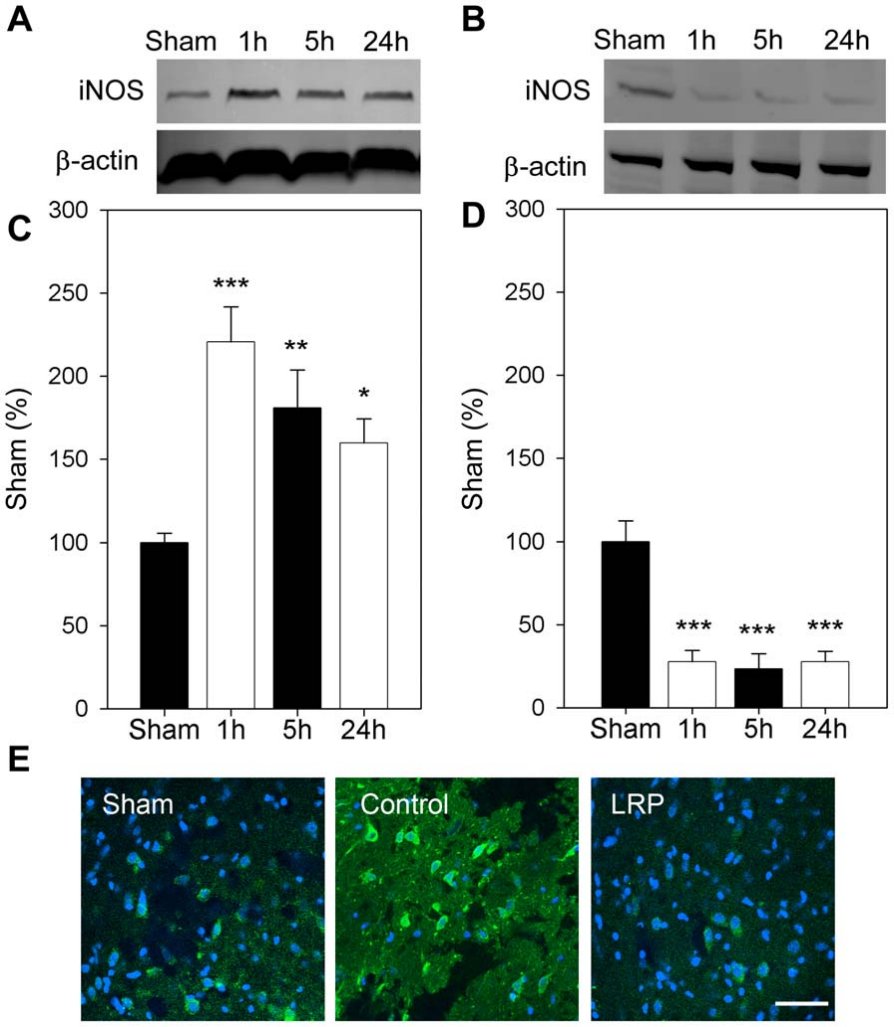
LRP blocked iNOS expression and nitrotyrosine after stroke. **A and C.** Representative protein bands of iNOS and quantitative results in the penumbra are shown for control ischemia. **B and D.** Representative protein bands of iNOS in the penumbra and quantitative results for the animals receiving control ischemia and LRP. iNOS was increased from 1 to 24 h after stroke in rats receiving control ischemia. LRP inhibited iNOS expression. *, **, *** vs. sham, *P*<0.05, 0.01. 0.001, respectively. n = 6/group. **E.** LRP blocked nitrotyrosine expression. Nitrotyrosine is a product of iNOS activities. Immunostaining suggested that nitrotyrosine expression was increased 24 h after stroke in control ischemic rats, and this was attenuated by LRP. Scale bar, 50 µM.

We also examined some critical molecules related to free radical production and inflammation, including iNOS and nitrotyrosine; nitrotyrosine is a product derived from iNOS activity. Western blot showed that iNOS protein levels were increased as early as 1 hour and lasted up to 24 hours, and its expression was blocked by LRP (Fig. 7A–D). Immunostaining also showed highly increased nitrotyrosine levels 24 hours post-stroke and LRP attenuated its expression (Fig. 7E).

## Discussion

This study contains several novel findings. First, we demonstrated that LRP offers long-term protective effects against focal ischemia in rats, as evidenced by the reduced size of brain injury measured 2 months post-stroke and improved performance during behavioral tests. Second, we provided more data to support the involvement of afferent nerve pathways in transferring protective signaling from the ischemic limb to the ischemic brain, as two afferent sensory nerve inhibitors, capsaicin and hexamethonium, blocked the protective effects of LRP. A recent study also showed that hexamethonium blocked the protective effects of limb preconditioning [4]. Third, we further showed that the galectin-9/Tim-3 pathway is involved in neuronal injury after stroke, and LRP blocked its overexpression. Last, we found that LRP inhibited edema formation, BBB permeability, and iNOS and nitrotyrosine production.

We offered solid evidence that LRP has chronic protective effects against stroke based on the measurement of brain injury and behavioral tests up to 2 months post-stroke. It is important to confirm this effect for clinical translation because several neuroprotectants, such as certain types of post-ischemic hypothermia [21] and rapid ischemic preconditioning [22], only transiently reduced infarct size. More recently, we also found that limb ischemic postconditioning, which was performed after reperfusion, reduced infarct size measured at 2 days but not at 1 month after stroke [11]. Nevertheless, in the current study, LRP not only reduced the loss of brain tissue measured 2 months after stroke, but also attenuated deficits in behavioral tests performed from 1 day to 2 months post-stroke, suggesting a long-term protective effect of LRP against stroke.

The afferent nerve systems appear to transfer the protective signaling from the preconditioned limb to the brain. Afferent neurons receive and transmit information from the peripheral organs or tissues to the central nervous system and contribute to the organism's ability to maintain homeostasis. In the case of ischemic limb preconditioning, repetitive ischemia and reperfusion resulted in the release of substances, such as adenosine and bradykinin [23] [24], which stimulate the afferent neurons that may transmit protective signaling to the brain. The afferent nerve system consists of peripheral fibers and endings along with neuronal bodies located in the spinal sensory ganglia that ascend to the brain stem and specific nuclei in the thalamus which, in turn, send information to the cerebral cortex. It is known that information from one side of the body can be sent to the opposite cortex in the primary sensory cortex and both sides of the secondary cortex [25]. In this study, both limb and brain ischemia were performed in the left side. We showed that capsaicin, which causes desensitization via its action on the peripheral fibers and endings of the afferent neurons [13], [14], blocked the protective effects of LPR. In addition, we found that hexamethonium, which inhibits the afferent neurons by blocking the ganglion [12], [24], [26], [27], also enlarged infarct size in animals treated with LRP. These two experiments suggest that protective information from the left limb can be sent to the same side of the brain cortex via the afferent neuronal pathways. Nevertheless, additional experiments should be conducted to more clearly demonstrate that remote preconditioning is transmitted to the brain by afferent innervation.

We showed that the galectin-9/Tim-3 pathway is involved in neuronal injury induced by cerebral ischemia and that LRP attenuated the expression of galectin-9 and Tim-3 in the ischemic brain, suggesting that the inhibition of this pathway may contribute to the protective effects of LRP. This is a novel pathway involved in immune modulation and inflammatory response [28]–[30]. It was originally indentified in T cells and subsequently in macrophages and dendritic cells. A recent study in mice showed increased mRNA levels of Tim-3 in the brain 3 days after stroke [31], but the cell types on which it was induced was not reported. In this study, we showed that protein expression of galectin-9 was increased as early as 5 hours after stroke, but Tim-3 levels were only increased 24 hours later. It seems that Tim-3 overexpression was induced after galectin-9, which is consistent with previous studies that galectin-9 is a trigger for Tim-3 activities. Since the major role of the galectin-9/Tim-3 pathway is to induce cell death, its induction in ischemic neurons might be attributable to ischemic neuronal injury. Nevertheless, galectin-9 was induced at 1 hour after stroke in the LRP group, which is earlier than that in the control group. Whether this early induction is beneficial or detrimental is not known. Despite this, we found that LRP inhibited increases in both galectin-9 and Tim-3 expression at 24 hours post-stroke, suggesting that inhibition of the galectin-9/Tim-3 pathway may be a target for stroke therapy.

Last, we found that LRP blocked increases in iNOS and nitrotyrosine expression. The induction of iNOS is critical for neuronal injury and inflammation under oxidative stress, including stroke [32]–[34]. In our study, iNOS protein expression was immediately upregulated after focal ischemia from 1 to 24 hours, and these increases were robustly blocked by LRP. Nitrotyrosine is a product of tyrosine nitration generated by ROS, a maker of NO-dependent products derived from iNOS, and its expression indicates cell damage and inflammation [35]–[37]. Thus, we also measured the expression of nitrotyrosine, which was also inhibited by LRP, corroborating its effects on iNOS.

In conclusion, we found that LRP provides long-term protection against focal cerebral ischemia, and it may transmit protective signaling through afferent nerve pathways. The inhibitive effects of LRP on edema formation, BBB permeability, the galectin-9/Tim-3 pathway, and ROS activities, may contribute to its protective effects against stroke.

## Materials and Methods

### Focal cerebral ischemia and remote preconditioning

Experimental protocols were approved by the Stanford University Administrative Panel on Laboratory Animal Care (Protocol #: APLAC 12642), and experiments were conducted in accordance with the guidelines of Animal Use and Care of the National Institutes of Health and Stanford University. Male Sprague-Dawley rats (250–350 g) were used. Anesthesia was induced by 5% isoflurane and maintained with 2–3% isoflurane during surgery and early reperfusion. Core body temperature was monitored with a rectal probe and maintained at 37°C during the entire experiment. Protocols for inducing focal ischemia and remote preconditioning are shown in Fig. 1. Animals in both the control ischemic and remote preconditioning groups received 90 min of anesthesia before stroke onset. Focal ischemia was induced as described previously [5], [8], [38]–[41]. The bilateral common carotid arteries (CCAs) were separated and occluded by aneurysms clips for 30 minutes, and the left distal middle cerebral artery (MCA) was cauterized above the rhinal fissure. To induce remote preconditioning, the left femoral artery was separated below the left groin ligament, occluded for 15 minutes and released for another 15 minutes, and repeated for 3 cycles. Stroke was performed immediately after remote preconditioning.

### Infarct size measurement

Rats were re-anesthetized with an overdose of isoflurane at the desired time after stroke, perfused intracardially with 100 ml of cold 10 mM sodium phosphate buffered saline (PBS; pH 7.4). The rats were then decapitated and the brains were rapidly removed and sectioned coronally at 2 mm intervals. Four sections were used for infarct measurement. As defined above, slices from rat brains 2 days post-stroke were incubated for 20 minutes in a 2% solution of 2,3,7-triphenyltetrazolium chloride (TTC) at room temperature and fixed by immersion in 4% paraformaldehyde (PFA) solution. Using a computerized image analysis system (NIH image, version 1.61), the area of infarction of each section was measured. Infarct size of the ischemic cortex was normalized to the non-ischemic cortex and expressed as a percentage, and an average value from the 4 slices was presented according to the formula: [(area of the cortex in the non-ischemic hemisphere – area of the normal cortex in the ischemic hemisphere)/area of the non-ischemic cortex]×100% [8], [38], [39].

Rat brains for those surviving 2 months were also sectioned into 4 blocks and fixed in 4% PFA/20% sucrose for 24 hours, frozen, sectioned into 30 µm slices on cryostat, and stained with cresyl/violet. The area of injured and lost ischemic cortex was traced under a microscope, scanned, measured and calculated as a percentage of the area of the intact, non-ischemic cortex according to the following formula: [(area of non-ischemic cortex - area of remaining ischemic cortex)/area of non-ischemic cortex]×100 [39].

### Behavior testing

Rats were randomly assigned into 3 groups for behavior tests: the sham surgery group received sham surgery without ischemia and preconditioning; the control ischemic group received stroke but without LRP; and the preconditioning group received both ischemia and LRP. We used a battery of standard behavior tests to quantify motor asymmetry caused by a unilateral cortical stroke as described in our previous studies [39], [41], [42]. All behavior tests were performed by a person who was blind to the experimental conditions. Most tests were performed before dMCA occlusion and then on days 1, 2, 3, 7, 10, 14, 21, 30, 37, 44 and 60 after dMCA occlusion.

The vibrissae-elicited forelimb placement test, which was used to detect forelimb placing against the edge of a table, was induced by gently brushing the rats' vibrissae on each side; the reflex was tested 10 times on each side per trial, and two trials occurred per test session. The percentage of vibrissae stimulations in which a paw placement occurred was calculated.

For the postural reflex test, the rat was placed on a table and the tail was held by one hand while the other hand gently pushed the animal's shoulder, moving it laterally ∼20 cm. The use of the forelimbs to resist the lateral movement was scored either 0, 1 or 2, with 0 being normal and 2 being no resistance, which was indicative of severe brain injury as defined in our previous study.

The home cage limb use test was performed after completing the other behavior tests. The animal was returned to its home cage, and the number of times the rat used its forelimbs to brace itself against the wall was counted; with separate counting for the ipsilateral, contralateral, or both forelimbs, until 20 such contacts were reached. The number of times out of 20 that the ipsilateral forelimb contacted was computed as a percentage using this formula: (ipsilateral+(both/2))/20×100%.

### Evaluation of BBB integrity

To examine whether LRP prevents BBB leakage, BBB integrity 48 hours post-stroke was studied using Evans blue [42]. Evans blue (4%, 2 ml/kg) was injected intravenously (i.v.) into ischemic rats, and the rats were perfused with heparinized saline solution 2 hours later. The brains were harvested, and the ischemic core and penumbra in the ipsilateral hemisphere and the contralateral cortex were dissected, weighed, homogenized and incubated in 500 µl formamide at 54°C for 2 hours. The solution was centrifuged at 12,000 g for 15 minutes, the supernatant was removed, and Evans blue was measured using spectrophotometry (absorbance at 620 nm) (Spectro Max 340, Molecular Devices, Sunnyvale, CA, USA). The amount of Evans blue was computed based on external standards in the same solvent (1–20 µg/ml) and expressed as per gram of tissue.

### Edema measurement

To examine the effect of LRP on edema, rats were randomly assigned to 3 groups: sham surgery rats without ischemia, ischemic rats without LRP and ischemic rats with LRP. Rats that survived 48 hours after stroke were euthanized for edema measurements using the wet-dry weight method [42]. Briefly, after euthanization, the ischemic and non-ischemic hemispheres from each block were separated, weighed for wet weight, baked at 90±2°C for 1 week, and weighed again for dry weight. Water content in brain tissues was calculated as: [1−(dry weight/wet weight)]×100%. The mean values from the left 6 blocks of ischemic hemisphere and from the right 6 blocks of non-ischemic hemisphere represent the water content in the ischemic and non-ischemic hemisphere, respectively.

### Drug injection

We used the afferent nerve blocker, capsaicin, to test whether the nerve pathway is involved in transferring protective signaling from the limb to the brain during remote preconditioning. Capsaicin was dissolved in 10% ethanol, 10% Tween-80 and 80% saline to a final concentration of 4 mg/ml. The method for capsaicin injection was modified from previous studies [13], [43].

To examine the effect of systemic injection of capsaicin on LRP, rats under isoflurane anesthesia were subcutaneously injected with capsaicin or vehicle for 4 consecutive days (n = 6/group): day 1, 12.5 mg/kg; day 2, 12.5 mg/kg, twice at 12 hour intervals; day 3, 25 mg/kg twice at 12 hour intervals; day 4, 25 mg/kg. To check the effectiveness of the capsaicin denervation, one drop of a 0.1 mg/ml solution of capsaicin was placed onto the eye of each rat and their protective movements were observed. All animals pretreated with capsaicin showed no wiping movements, thus confirming functional denervation of the capsaicin-sensitive nerves. Focal ischemia and LRP were performed 2 weeks after the last capsaicin injection. The operator performing the surgery was blind to the rats' condition. All rats receiving drug and vehicle treatments were sacrificed 2 days after stroke for infarction measurement.

To examine if local application of capsaicin also affects LRP, capsaicin was applied to the left thigh nerve of rats. In brief, rats were anesthetized and the left thigh nerve was exposed. Approximately 1-cm-long segments of left nerve were isolated with Parafilm and small pieces of gelfoam moistened with a capsaicin solution (1%, 100 µl) were wrapped around the nerve for 30 min. The capsaicin-exposed area was then flushed with saline and the wound was closed. In some control animals, the left nerve was similarly treated with saline. LRP and focal ischemia were performed 4 days later.

We further used the ganglionic blocker, hexamethonium, which blocks the afferent nerve pathways, to test whether the afferent nerves transfer protective signaling from the preconditioned limb to the brain. Hexamethonium was dissolved in saline and injected (i.v) (20 mg/kg) into rats 30 minutes before LRP. Thereafter, stroke was induced and animals were euthanized 2 days later for infarct size measurement.

### Immunofluorescence staining and confocal microscopy [39]

Rats were transcardially perfused with ice-cold PBS (pH 7.4) and 4% PFA solution. Brains were post-fixed in 4% PFA solution and cytoprotected in 20% sucrose at 4°C overnight. We stained cryostat sections (40 µm) with antibodies against Tim-3 (1∶100; Santa Cruz Biotechnology; sc-30326), Galectin-9 (1∶50; Santa Cruz Biotechnology; sc-19292), and nitrotyrosine (1∶50; Millipore (Chemcon); 92590). Neurons were stained with microtubule-associated protein 2 (MAP-2) (1∶200; Sigma; M4403). Double staining of Tim-3 and MAP-2 was performed consecutively with their primary and secondary antibodies, respectively. Fluorescent-stained sections were analyzed by confocal microscopy.

### Western blot

Rats were randomly assigned into different groups, euthanized at 1, 5, 24, and 48 hours (n = 6/group), and rat brains were harvested for immunostaining and western blot at different time points as described previously [39], [41]. Samples were lysed with RIPA buffer (10 mM TRIS, 140 mM Nacl, 1% Triton, 1% Na-deoxycholate, 0.1% SDS, 0.5 mM phenylmethylsulfonyl fluoride (PMSF) and supplemented with cocktail inhibitors; Roche). Extracts were homogenized and insoluble debris removed by centrifugation at 6000 g for 10 minutes at 4°C. Protein concentration in the resulting supernatants was calculated using a Pierce protein assay kit according to the manufacturer's instructions (Pierce, IL). Equal amounts of protein samples (13 µl) were loaded, separated using 4–15% SDS-polyacrylamide gel (Bio-Rad Laboratories, Hercules, CA) electrophoresis and then transferred to Trans-Blot nitrocellulose membrane (BioRad, CA). Membranes were scanned using Typhoon trio (GE Healthcare). We used primary antibodies against Tim-3 (1∶500; Santa Cruz Biotechnology; sc-30326), Nitrotyrosine (1∶500; Millipore (Chemcon); 92590), iNOS (1∶10000; BD Biosciences; 610431), and β-actin (1∶10000; Sigma; A3854).

### Statistical Analysis

One-way analysis of variance (ANOVA) was used to compare each group on infarct size followed by Fisher's least square difference *post hoc* test when there were more than 3 groups; t-test was used to compare infarct size when only 2 groups were studied. For behavioral tests, one-way repeated measures ANOVA was used to compare tests at different time points in the same group, and two-way ANOVA was used to compare between LRP and control ischemia, followed by the Student–Newman–Keuls test. Tests were considered significant at *P*-values<0.05. Data are presented as mean ± s.e.m.
